# Acid-Resisting Mackintosh

**Published:** 1911-09-23

**Authors:** 


*To the Editor of* The Hospital.

### ACID-RESISTING MACKINTOSH.

*To the Editor of* The Hospital.

Sir,—I have seen in your paper a letter with inquiry for good mackintosh sheeting for a bed-ridden case. I can recommend that supplied by Messrs. J. H. Pontifex, Ltd., 99 Buckingham Palace Road, as being most reliable. I have always found their goods satisfactory.

Regent's Park, September 16. A.

